# Erythrocyte nicotinamide adenine dinucleotide concentration is enhanced by systematic sports participation

**DOI:** 10.1186/s13102-024-00999-y

**Published:** 2024-10-15

**Authors:** Barbara Pospieszna, Krzysztof Kusy, Ewa Maria Slominska, Jacek Zieliński, Monika Ciekot-Sołtysiak

**Affiliations:** 1 Department of Athletics, Strength and Conditioning, Poznan University of Physical Education, Królowej Jadwigi 27/39, Poznan, 61-871 Poland; 2 Department of Biochemistry, Medical University of Gdansk, Gdansk, Poland

**Keywords:** Nicotinamide adenine dinucleotide phosphate, Endurance, Sprint, Master athletes, Elderly

## Abstract

**Background:**

Nicotinamide adenine dinucleotide (NAD+), nicotinamide adenine dinucleotide phosphate (NADP+), and their reduced forms (NADH and NADPH) are the vital cofactors for most cellular oxidation/reduction reactions and therefore influence most critical pathways in cellular metabolism. This study aimed to predict the trends of age-related changes in erythrocyte NAD+ and NADP+ concentrations in elite athletes compared to untrained controls and to assess whether life-long physical training stimulates favorable adaptations in erythrocyte NAD(P)+ concentrations.

**Methods:**

Erythrocyte concentrations of NAD+ and NADP+ were measured in 68 elite endurance runners (20–81 years), 58 elite sprinters (21–90 years), and 62 untrained individuals (20–68 years). Linear regression analyses were performed to estimate longitudinal relationships and cross-sectional rates of change between age and erythrocyte NAD+ and NADP+ levels. One-way analysis of variance was used to determine differences between the studied groups.

**Results:**

In all three groups, the erythrocyte NAD+ and NADP+ concentrations significantly decreased with advancing age, suggesting gradual deterioration of NAD-related regulatory functions in older individuals. However, the concentration of erythrocyte NAD(P)+, regardless of age category, was higher in the athletic groups compared to less active controls.

**Conclusions:**

Our research shows that systematic sports participation, especially of a sprint-oriented nature, can be treated as a natural and effective strategy promoting cellular NAD(P)+ anabolism and thus cells’ energy and redox metabolism.

**Trial registration:**

The study was retrospectively registered in the clinical trials registry on 2021-11-09 (NCT05113914).

## Background

The pyridine nucleotide family (NAD(P)+) comprises nicotinamide adenine dinucleotide (NAD+), its reduced form NADH, and those two molecules after phosphorylation: NADP+ and NADPH. The level of NAD(P)+ and its metabolome − known also as the NADome − appears to be of special importance, as it provides an important link between signaling and metabolism [1–4]. For many years, scientific interest focused on the nicotinamide adenine dinucleotide family covered the explanation of (1) biosynthetic pathways and biochemistry of its turnover; (2) the physiological functions of NAD(P)+ in various human cell cultures and its main compartments; and (3) the integrated role of NAD(P)+ in human metabolism [1, 5, 6]. Recently, the nicotinamide adenine dinucleotide has been treated as a critical molecule in hundreds of metabolic pathways (e.g., energy production, cellular metabolic sensory pathways, redox reactions, inflammation, and gene expression regulation) [7]. Therefore, much effort has been made to gain a deeper understanding of the NAD+ metabolome and the role of modulating NAD + metabolites [8]. On the other hand, the cellular and plasma concentrations of NAD+ significantly decrease during chronological aging [1]. As NAD(P)+ serve as coenzymes in most oxidoreductase enzymes [5], the decline in its concentration is paralleled by age-associated changes in oxidative stress levels [9]. Moreover, negative alterations in NAD(P)+ homeostasis can be found in nearly all age-related diseases, e.g., cardiovascular, cerebrovascular, and neurodegenerative diseases, immune dysfunction, obesity, and cancer [1, 3, 5, 8, 10, 11]. The underlying mechanism of all these clinical disorders is linked to either inadequate energy production, inflammation, elevated levels of oxidative stress, or defective gene expression; hence, it is directly reliant on the concentration of NAD(P)+. Thus, there is a great interest in discovering ways to manipulate the magnitude of NAD+ synthesis or catabolism, finding NAD+ boosters, or other ways to prevent age-related NAD+ decline. From many attempts to establish efficient strategies to promote cellular NAD+ anabolism, the additional administration of NAD+ precursors, e.g., nicotinamide riboside and nicotinamide mononucleotide [2, 12, 13] or sirtuin-activating compounds, such as resveratrol [14], seems to be the most promising.

Erythrocytes (RBCs) are unique, simplified cells of great biological importance. The key erythrocyte biochemical functions include energy production (glycolysis and the pentose phosphate pathway), redox metabolism, oxygen metabolism, purine metabolism, and membrane transport [15]. Because of the lack of mitochondria and nuclei, these cells are entirely dependent on anaerobic glycolysis to produce sufficient levels of adenosine triphosphate (ATP), and many metabolic pathways typical for mammalian cells do not occur. As humans age, the main aspects of erythrocyte metabolism and physiology (e.g., speed of glucose influx, rate of glycolysis, stability of cells’ energy charge, osmoregulation, and electroneutrality) are considerably disturbed. These changes are associated with increased immunosenescence, inflammation, and oxidative stress [8, 15]. Previous research has shown age-related decreases in the levels of several erythrocyte molecules and key enzymes, including the concentrations of the pyridine nucleotide family [3, 9, 11, 16–18].

Various forms of physical activity can slow down or even prevent the negative course of age-dependent decline in several biochemical and physiological functions. Compared to untrained individuals, athletes usually have greater mental, physical, and functional capacity. Within the structural and biochemical activity-related adaptations, athletes have been shown to have higher concentrations of hormones (e.g., erythropoietin, adrenaline, cortisol, testosterone, growth hormone, etc.) and increased hematopoietic bone marrow activity, which directly converts into a greater number of reticulocytes and younger erythrocytes [19–21]. A higher hemoglobin content and increased membrane stability result in greater oxygen affinity, better osmotic resistance, and enhanced antioxidant protection [20, 22, 23]. Few studies have shown that physical training in middle-aged and elderly people leads to increases in muscle NAD+ and NADH concentrations and therefore enhances muscle mitochondrial adaptation [24–27]. However, to the best of our knowledge, there is a lack of research concerning the erythrocyte NAD(P)+ concentration and physical training. One study revealed that the concentrations of NAD+ and NADP+ in RBCs do not change after a single exercise test with progressively increased intensity [28]. The influence of NAD(P)+ in erythrocytes is practically limited to anaerobic energy production and preserving the cells’ antioxidant potential [15]. Our previous studies showed that, compared with recreationally trained controls, athletes have significantly higher erythrocyte energy potential, manifested by higher concentrations of erythrocyte adenosine triphosphate and guanosine triphosphate (GTP) [29–31]. We have also shown that the level and rate of age-related adenylate and guanylate decline depend on the type of training regime.

Addressing these concerns, this study aimed to evaluate the levels and predicted rates of age-related changes in erythrocyte NAD+ and NADP+ in elite athletes compared to untrained controls. We hypothesized that (i) the erythrocyte concentrations of NAD+ and NADP+ are greater in athletes than in untrained controls regardless of age and that (ii) the level and rate of the age-related decrease in the concentrations of NAD+ and NADP+ depend on the type of used training model.

## Methods

This study was a part of larger research involving two hundred and six healthy, non-smoking men between the ages of 20 and 90. There were two track and field athletic groups: endurance runners (EN; 86 men aged 20–81 years) and sprint-trained athletes (SP; 58 men aged 21–90 years), and a control group (CO; 62 untrained individuals aged 20–68 years). Inclusion and exclusion criteria, along with participant’s recruitment and study procedures, have been described in detail in our previous publications [29–31]. All study procedures were approved by the local ethics committee (1079/12). The study was registered in the Clinical Trial Registry (NCT05113914). Each participant was informed of the study’s testing procedure, purpose, and risks, and provided written informed consent. After basic anthropometric measurements (height and weight), blood collection, a light standardized breakfast, and subsequent rest, well-rested participants underwent an incremental running test until volitional exhaustion on a mechanical treadmill (h/p/cosmos Sports & Medical GmbH, Nussdorf-Traunstein, Germany). During the test cardiorespiratory parameters were continuously measured by an ergospirometer (MetaMax 3B-R2, Cortex Biophysics GmbH, Leipzig, Germany) with a Polar Bluetooth Smart H6 (Polar Electro Oy, Kempele, Finland) heart rate monitor. Individual values of maximal oxygen uptake ($\:\dot{V}$O_2_max) were assessed according to the best agreement between two independent observers.

In the venous blood samples the basic hematological indices were immediately determined using the Mythic^®^ 18 hematology analyzer (Orphée, Geneva, Switzerland). Our previous publications [29–31] have also described in detail the RBC sampling and analysis procedure. Within 3 min after blood collection, the erythrocytes were separated and washed three times. The resulting erythrocyte pellet was then deep-frozen at -80 °C until biochemical analysis of NAD+ and NADP+ concentrations. The measurements were performed using high-performance liquid chromatography (HPLC) with UV‒VIS detection (Merck-Hitachi/Agilent, Japan/USA).

All the statistical analyses were performed using Statistica 13.3 software (StatSoft, Inc., Tulsa, OK) and Python 3.12.2 programming language with relevant statistical & data visualization libraries. The sample size of 36 participants per group was a priori calculated to ensure the significant detection of differences and correlations (G*Power software; Heinrich-Heine-Universität, Düsseldorf, Germany). The normal distribution of the analyzed variables (Shapiro‒Wilk test) allowed parametric tests to be used in later calculations. To identify most correlated variables in the data, each variable’s values have been converted into Z-scores and the correlation matrix was then generated to examine the linear relationships between the standardized variables, using Pearson’s correlation coefficient. Linear regression analyses and regression equations (slopes of regression lines) were performed to estimate longitudinal relationships and cross-sectional rates of change between age and erythrocyte NAD+ and NADP+ levels. One-way analysis of variance (ANOVA) with a post-hoc Bonferroni correction was used to determine differences between the studied groups. The equal slopes test with the post-hoc Bonferroni correction was used to determine differences between the slopes of the regression lines. The level of significance was set at 0.05. The statistical power of the ANOVA results for NAD+ and NADP+ was 1.0.

## Results

All values are presented as the mean ± standard deviation (SD). Table 1 presents the study participants’ somatic and exercise characteristics and hematological and biochemical indices measured in their blood. Like the athletes’ training history and current training volume, the mean participants’ age did not significantly differ between the groups. Endurance runners were lighter than sprinters and controls, were smaller than controls, and had a lower BMI than sprinters. The maximal oxygen uptake differed between the groups; it was lowest in the control group and highest in endurance-trained athletes. There were no differences in the hematological indices between the athletic groups; however, the hemoglobin concentration was significantly higher in the EN compared to CO, and the mean corpuscular hemoglobin concentration was greater in the SP group than in the CO group. The erythrocyte concentrations of NAD+ and NADP+ significantly differed between the groups and were highest in the SP group, lower in the EN group, and lowest in the CO group.

**Table 1:** Somatic, exercise and hematological characteristics (mean ± SD) of endurance runners, sprinters and controls

Variable	EN	SP		CO	*p*	Effect size (η^2^)
Age	46.41 ± 15.24	47.33 ± 19.07		44.08 ± 15.12	0.527	0.006
Training history (years)	26.7 ± 14.4	28.6 ± 18.8			0.552	0.411
Weekly training (hours/week)	9.1 ± 3.5	8.7 ± 3.2			0.547	0.365
BM (kg)	72.37 ± 6.97^†,‡^	77.57 ± 8.42^§^		75.97 ± 6.09^§^	< 0.001*	0.090
BH (cm)	177.27 ± 5.58^‡^	179.42 ± 8.22		179.95 ± 5.14^§^	0.024*	0.036
BMI (kg/m^2^)	23.03 ± 1.97^†^	24.07 ± 1.73^§^		23.48 ± 1.40	0.003*	0.057
$\:\dot{V}$O_2_max (ml/kg/min)	58.60 ± 8.58^†,‡^	48.15 ± 8.46^§,‡^		41.66 ± 5.64^§,†^	< 0.001*	0.468
Hb (g/l)	15.37 ± 1.02^‡^	15.16 ± 0.83		14.88 ± 0.73^§^	0.005*	0.052
HCT	0.45 ± 0.03	0.44 ± 0.02		0.44 ± 0.02	0.041*	0.031
MCHC (g/dl)	34.36 ± 2.09	34.82 ± 1.59^‡^		33.86 ± 1.42^†^	0.014*	0.041
NAD+ (µmol/L RBC)	73.19 ± 10.88^†,‡^		85.21 ± 10.94^§,‡^	64.64 ± 10.30^§,†^	< 0.001*	0.353
NADP+ (µmol/L RBC)	51.05 ± 7.134^†,‡^		57.80 ± 6.457^§,‡^	43.64 ± 8.153^§,†^	< 0.001*	0.359

* – significant between-group differences as indicated by one-way ANOVA (*p* < 0.05);

† – significantly different from sprint-trained athletes; ‡ – significantly different from untrained controls; § – significantly different from endurance-trained athletes

Legend: BM ‒ body mass, BH – body height, BMI ‒ body mass index, $\:\dot{V}$O_2_max – maximal oxygen uptake, Hb ‒ hemoglobin, HCT – hematocrit, MCHC – mean corpuscular hemoglobin concentration, NAD+ – nicotinamide adenine dinucleotide, NADP+ – nicotinamide adenine dinucleotide phosphate, EN – endurance runners (*n* = 86), SP – sprinters (*n* = 58), CO – controls (*n* = 62)

**Fig. 1:**
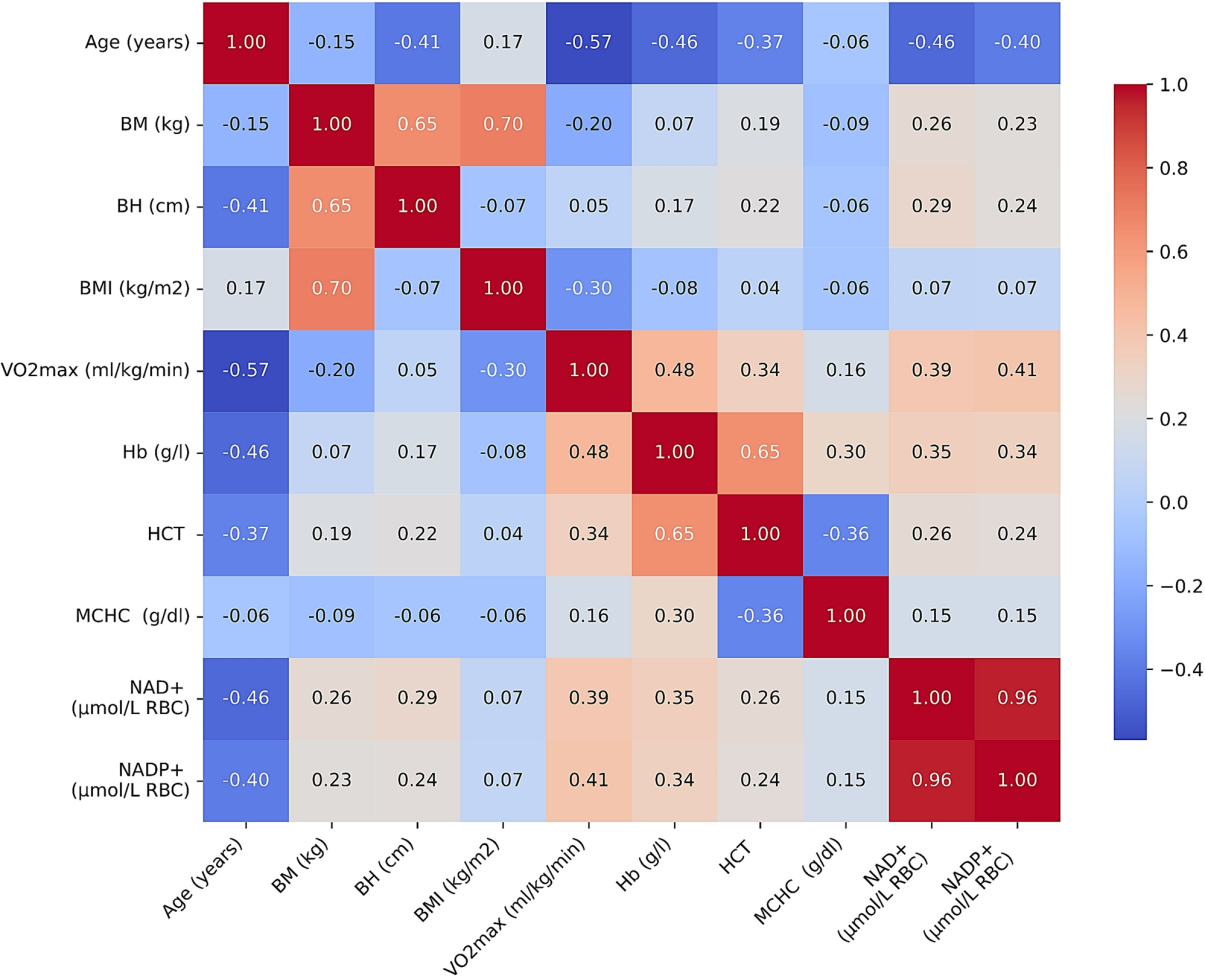
The overall correlation matrix across analyzed variables. *Notes* BM, body mass; BH, body height; BMI, body mass index; $\:\dot{V}$O_2_max, maximal oxygen uptake; Hb, hemoglobin; HCT, hematocrit; MCHC, mean corpuscular hemoglobin concentration; NAD+, nicotinamide adenine dinucleotide; NADP+, nicotinamide adenine dinucleotide phosphate

The analysis of the physiological and biochemical variables, based on Pearson correlation coefficients, revealed several key relationships (Fig. 1). A strong positive correlation was observed between body mass (BM), body height (BH), and BMI, underscoring their inherent interdependence. Similar correlations were found between hematological (Hb, HCT) and metabolic (NAD+, NADP+) variables. Age showed a significant inverse correlation with $\:\dot{V}$O_2_max, hemoglobin content, and NAD(P)+ concentrations indicating that both aerobic capacity and metabolic efficiency tend to decline with age. Additionally, a positive correlation was observed between $\:\dot{V}$O_2_max and the concentrations of NAD+ and NADP+ suggesting the positive impact of physical capacity on cellular energy and redox metabolism (See Fig. 1).

In all three groups, most variables significantly decreased with age, as predicted by linear regression. A medium effect size was observed for hemoglobin (*p* ≤ 0.001; r^2^ = 0.23–0.27), whereas a large effect size of age-related decline was achieved for $\:\dot{V}$O_2_max (*p* ≤ 0.001; r^2^ = 0.62–0.77).

**Fig. 2:**
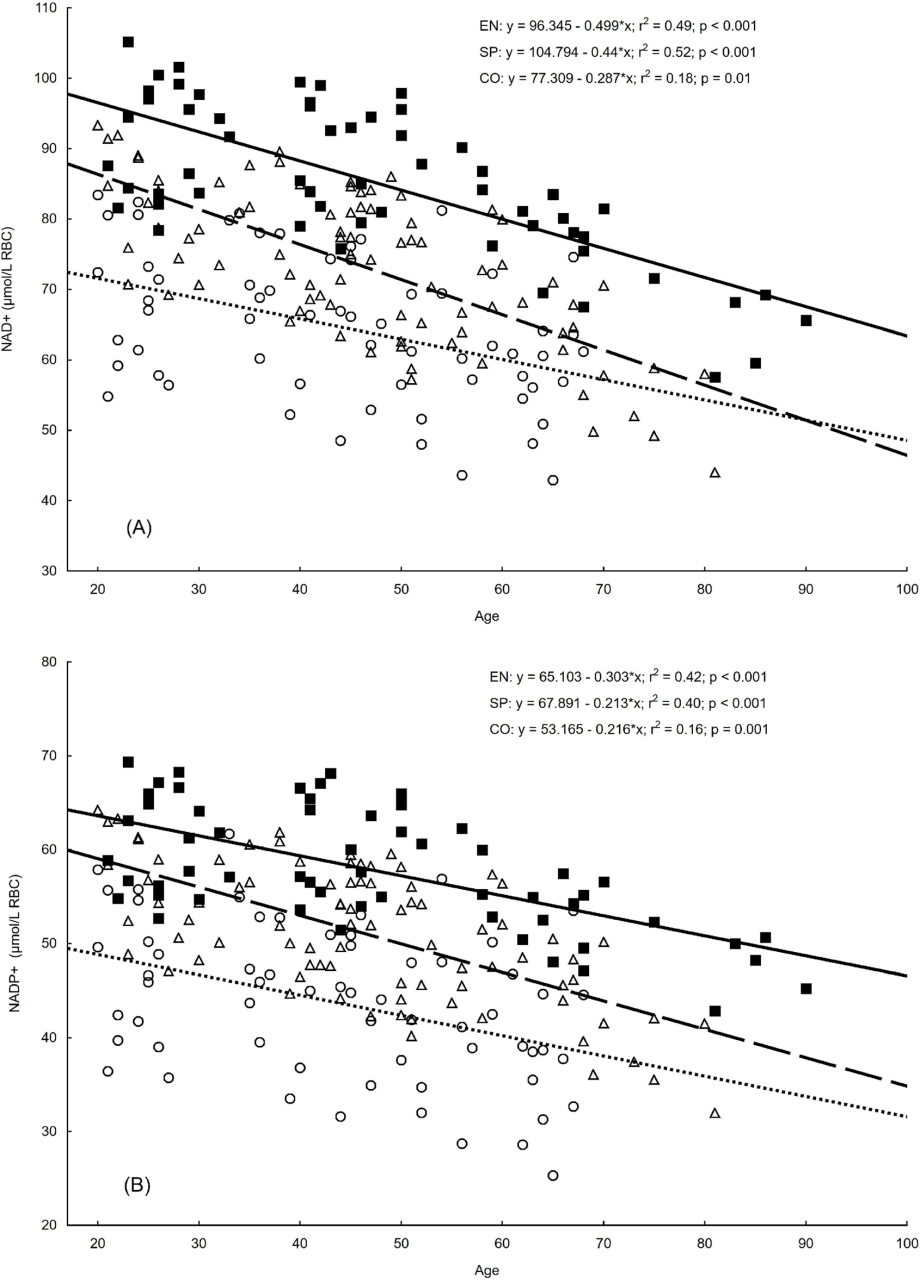
Relationships between age and the erythrocyte concentrations of nicotinamide adenine dinucleotide and nicotinamide adenine dinucleotide phosphate. *Notes* (**A**) NAD+, nicotinamide adenine dinucleotide; (**B**) NADP+, nicotinamide adenine dinucleotide phosphate; (Δ, – – –), endurance runners (EN; *n* = 86); (■, ——) sprinters (SP; *n* = 58); (○,….), controls (CO; *n* = 62)

In all the groups, the linear relationships between age and the resting concentrations of erythrocyte NAD+ and NADP+ were significant (Fig. 2). In both athletic groups, the coefficients of determination were at a modest level (r^2^ = 0. 40–0.52), whereas in the control, the correlation was very low (r^2^ < 0.20).

As shown in Table 2, the predicted rate of age-related decline in the concentration of erythrocyte NAD+ was highest in the EN group, lower in the SP group, and lowest in the CO group. The cross-sectional decline in the NADP+ concentration was the smallest in the control group and the greatest in endurance-trained athletes. Bonferroni post-hoc correction revealed significant differences between all slopes of the regression lines for both variables.

**Table 2:** Absolute and relative cross-sectional changes (rates of age-related decline) in the concentrations of erythrocyte NAD+ and NADP+

Variable (µmol/L RBC)	EN		SP		CO		Differences of slopes	
	Absolute	Relative	Absolute	Relative	Absolute	Relative	*p*	Effect size (η^2^)
NAD+	-0.499^†,‡^	-5.778	-0.440^§,‡^	-4.584	-0.287^§,†^	-4.016	< 0.001*	0.168
NADP+	-0.303^†,‡^	-5.128	-0.213^§,‡^	-3.352	-0.216^§,†^	-4.422	< 0.001*	0.103

* – significant differences between the slopes of the regression lines (*p* < 0.05)

† – significantly different from sprint-trained athletes; ‡ – significantly different from untrained controls; § – significantly different from endurance-trained athletes

Legend: NAD+ – nicotinamide adenine dinucleotide, NADP+ – nicotinamide adenine dinucleotide phosphate, EN – endurance runners (*n* = 86), SP – sprinters (*n* = 58), CO – controls (*n* = 62), absolute – units of measure per year, relative – percentage per decade

## Discussion

The NAD+ nucleotide−as the precursor of all the pyridine nucleotide family−is a key cellular component of great importance. By affecting hundreds of crucial metabolic pathways and most physiological functions, it provides an important link between signaling and metabolism [1, 2]. Since NAD+ deficiency is linked to various pathological states and its concentration is negatively dependent on age, numerous justified attempts have been undertaken to promote cellular NAD+ anabolism, especially the additional administration of NAD+ precursors [3, 5, 8, 13, 14, 32]. Our goal was to determine whether life-long systematic sports participation−known for its beneficial health influence−naturally enhances the erythrocyte concentration of NAD(P)+. The main finding of our study is that, regardless of age category, NAD(P)+ levels remain higher in athletes (especially in sprinters) compared to untrained controls. In all the study groups, the direction and magnitude of the predicted age-related changes in erythrocyte NAD(P)+ were substantially similar. However, the rate of decline was significantly lower in the SP compared to EN and CO groups. The highest concentrations of NAD+ and NADP+ strongly suggest that sprint-oriented training efficiently preserves NAD-related erythrocyte functions across a wide age range.

Cellular NAD+ metabolism involves a wide spectrum of biochemical pathways and cellular processes, even when it is highly compartmentalized to specific subcellular pools: the cytoplasm, mitochondria and nucleus. As a simplified cell lacking nucleus and mitochondria, the erythrocyte is an ideal research model. Although, there are many studies concerning NAD+ decreases in muscle, liver, skin, and other tissues [1, 3, 27, 33, 34], there exist only a few concerning RBCs.

The abundance of erythrocyte NAD+ and NADP+ is much greater than that of their reduced forms, so we focused on these forms of pyridine nucleotides. In a quantitative blood metabolomics analysis of 15 young and 15 elderly individuals, Chaleckis et al. [11] reported that the concentrations of NAD+ and NADP+ showed remarkable age-related changes. There was a significant difference in the levels of NAD+ (*p* = 0.046) and NADP+ (*p* = 0.022) between young and elderly, suggesting a decrease in redox metabolism in elderly RBCs. However, the limitation of that research was the comparison of the results of only two small groups of individuals with a large age gap (29 ± 4 y vs. 81 ± 7 y of age). In our research, we focused on revealing the predicted rates of age-related changes in erythrocyte NAD(P)+ and showing their absolute and relative changes with advancing age. Although NAD+ is primarily an intracellular nucleotide, some considerable amounts of that molecule can also be found in the plasma. Using a modified LS‒MS approach, Clement et al. [8] observed the similar trend of age-related NAD+ and NADP+ decline in the blood plasma of 29 individuals of both sexes, who were divided into three age groups: 22.78 y (*n* = 9), 52.8 y (*n* = 10), and 76.9 y (*n* = 10).

Another study on age-related changes in the erythrocyte metabolome confirmed the progressive decline in the NAD+ concentration and linked it mainly with the downregulation of the Embden–Meyerhof pathway. However, metabolic alterations in the pentose phosphate pathway and the Luebering‒Rapoport shunt were also observed in this study [3]. In one of the early studies, Zerez et al. [17]−using pyruvate kinase-deficient human erythrocytes−proved that the decreased ATP regeneration is directly related to impaired NAD+ biosynthesis. Similar findings on the decreased ability of aging erythrocytes to metabolize glucose and therefore efficiently produce ATP have been presented in other studies [18, 29].

NAD+ concentrations fluctuate in a circadian manner [4, 33] and change under various environmental influences. Studies concerning cellular NAD(P)+ concentrations have shown that NAD(P)+ concentrations decrease during aging and senescence [2, 9], and under conditions decreasing life or health span, especially high-fat diets [35, 36]. In contrast, NAD(P)+ concentrations tend to increase under conditions known to increase life or health span, associated with caloric restriction (such as fasting or glucose deficiency) or exercise [36–38]. To date, many studies concerning fluctuations in the NAD(P)+ concentration of skeletal muscle under different exercise treatments have been conducted, primarily covering mitochondrial adaptations [24–27]. Thus, studying the adaptation of NAD(P)+ to exercise in mitochondrion-deficient erythrocytes is highly interesting. A single exercise test with progressively increased intensity on a cycloergometer was proven to be an inadequate stimulus to significantly disturb the erythrocyte energetic equilibrium [28]. Single maximal exercise performed in twenty-two healthy young (age = 21.9 ± 2.33 years), recreationally trained ($\:\dot{V}$O_2_max = 45.8 ± 4.11 ml·kg-1·min-1) male volunteers did not cause any changes in erythrocyte adenine, guanine or pyridine nucleotide concentrations. We have previously shown that using physical training, significant adaptations in erythrocyte energetics can be obtained over a longer period (1 year), but only through periodized training in competitive athletes [30]. In our other research, we have presented that although the energetic condition of erythrocytes (measured by the negative changes in the concentrations of both adenylate and guanylate metabolites) gradually decreases between 20 and 90 years of age, the concentrations of particular metabolites are more advantageous in highly trained athletes compared to less active controls [29, 31]. The results of this study add to the current knowledge that lifelong sports participation also results in higher concentrations of NAD+ and NADP+ with advancing age.

Longitudinal participation in various forms of physical activity positively affects blood volume and composition, predominantly by modulating hematopoietic bone marrow activity and elevating the concentration of hormones, subsequently stimulating RBC production and release [19–21]. Frequent involvement in high-intensity exercise−and resulting extreme homeostasis disturbances−lead to a higher rate of hemolysis and a shorter RBC lifespan [39]. The consequential faster RBC turnover causes a positive shift toward the younger RBC population, with higher amounts of reticulocytes [20, 22, 23]. Before the transition into erythrocytes, reticulocytes circulate in the bloodstream for approximately 1–2 days [40]. Compared with mature erythrocytes, reticulocytes still contain mitochondria and thus effectively produce energy through oxidative phosphorylation. Moreover, they exhibit increased glycolytic and pentose phosphate flux and display higher activity of glycolytic enzymes (e.g., hexokinase, aldolase, and pyruvate kinase) [16, 41]. Therefore, a greater number of reticulocytes and a higher proportion of young RBCs in the bloodstream indicate a higher erythrocyte energy status and more effective production of pyridine nucleotides for fighting oxidative stress and maintaining osmotic balance and electroneutrality [15]. It seems probable that the higher erythrocyte energy status (highest ATP and GTP concentrations [29, 31]) and higher concentrations of NAD(P)+ in sprinters of all ages, compared to endurance athletes and recreationally trained controls, originate from more frequent exposure to high-intensity exercise and thus better metabolic adaptation.

Interestingly, the rate of age-dependent decline in NAD(P)+ in endurance athletes was more pronounced than that in SP and CO. In younger individuals, the NAD(P)+ concentration was significantly higher in endurance athletes compared to controls. At older ages, however, the concentrations of both molecules are very similar in the EN and CO groups, whereas in SP, they remain at a much higher level. This phenomenon requires deeper examination; however, it might be explained by the typical change in the training intensity of older endurance athletes toward less intensive but longer sessions. At the same time, controls and sprinters usually do not change the intensity of their daily activities that much. This more pronounced decrease in pyridine nucleotides in EN, compared to CO and SP, is paralleled by a more evident decrease in the concentration of GTP [31], but not ATP [29], in older endurance athletes, as shown in our previous papers. This relationship might result from ATP production being maintained by both glycolysis and the pentose phosphate pathway, as those two biochemical pathways share some intermediates and enzymes to generate ATP more efficiently [15]. Erythrocyte GTP production, however, is partly dependent on the NAD+ supply, as the pathway from inosine monophosphate to guanine monophosphate is ATP- and NAD+-dependent [42].

Given that negative alterations in NAD+ homeostasis are linked to aging and that they can be found in nearly all age-related diseases, there is a great interest in discovering ways to manipulate either the magnitude of NAD+ synthesis or its catabolism. There have been many attempts to find ways to prevent age-related NAD+ decline and thus improve health and life span, with a particular focus on discovering marketable NAD+ boosters.

First, three main NAD+ precursors were analyzed, namely, tryptophan, nicotinic acid, and nicotinamide, but in vivo, they all were considered rather poor NAD+ precursors, and due to their side effects, are rarely used in clinical practice [13]. Recently, the administration of nicotinamide riboside and nicotinamide mononucleotide (the intermediates in the NAD+ salvage pathway) gained scientific interest, as these compounds seem to effectively increase NAD+ biosynthesis and have a favorable influence on the pathology of some age-related disorders [5, 32, 43]. In parallel, several promising trials have been performed with sirtuin-activating compounds, such as resveratrol [1, 14]. However, many studies have failed to provide any measurable physiological improvements after such supplementation [1, 5, 7, 14, 44]. One of the most likely reasons for supplements’ ineffectiveness might be the inclusion of healthy individuals in the trials, as the studies including only carefully prescreened patients (older, suffering from metabolic disease, cancer, cognitive decline, etc.) tend to achieve more positive results. There are also findings showing that exogenously administered nicotinamide riboside in young rodent populations might even lead to adverse physiological effects [45]. It is possible that redox supplementation is beneficial only in individuals with considerable antioxidant deficiencies and that in healthy individuals, well-designed physical training and/or calorie restriction may prove to be more effective and concurrently deprived of various metabolic side effects.

The main limitation of this study is that only the concentrations of nicotinamide adenine dinucleotides in the oxidized forms (NAD+ and NADP+) were analyzed. In future research, analyzing the ratios of non-phosphorylated forms (NAD and NADH) and phosphorylated forms (NADP and NADPH) simultaneously or even the whole metabolome under different kinds of physical training would be more informative. As we included only healthy men in the study, our findings are limited to only one sex. Given that gender may impact metabolism, however, it doesn’t impact the RBCs metabolic profile [3], it would be interesting to determine whether women respond to physical training similarly.

## Conclusions

In conclusion, systematic sports participation, especially sprint-oriented, can be treated as a natural and effective strategy promoting cellular NAD+ anabolism and thus cells’ energy and redox metabolism. Increasing amounts of data convincingly indicate that exercising with high or even maximal intensities is more effective than exercising with moderate intensity. A higher exercise intensity results in greater metabolic disturbance and subsequent adaptive reactions. The results of our study may convince kinesiology and medical specialists to rethink the current state of knowledge and prescribe more training sessions of sprint-oriented character in the future.

## Data Availability

The dataset that support the findings of this study is available in the RepOD repository at 10.18150/C27DZ1.

## Abbreviations

NAD(P)+: Pyridine nucleotide family
NAD+: Nicotinamide adenine dinucleotide
NADP+: Nicotinamide adenine dinucleotide phosphate
NADH: Nicotinamide adenine dinucleotide hydrogen
NADPH: Nicotinamide adenine dinucleotide phosphate hydrogen
RBC: Erythrocyte, red blood cell
ATP: Adenosine triphosphate
GTP: Guanosine triphosphate
$\:\dot{V}$O_2_max: Maximal oxygen uptake
